# Perceived financial and health threats and wellbeing: the role of personal control in different life domains

**DOI:** 10.3389/fpsyg.2025.1539794

**Published:** 2025-10-31

**Authors:** Brianda Canal-Serantes, Ginés Navarro-Carrillo, Inmaculada Valor-Segura

**Affiliations:** ^1^Department of Social Psychology, Faculty of Psychology, University of Granada, Granada, Spain; ^2^Mind, Brain and Behavior Research Center, University of Granada, Granada, Spain

**Keywords:** personal sense of control, control in different life domains, financial threat, health threat, wellbeing, psychological distress

## Abstract

**Introduction:**

Contexts of heightened economic or health instability present a threat to perceived personal control and wellbeing. Although perceived personal control has been fundamentally postulated as a unitary category, there is some evidence suggesting that levels of perceived personal control might vary throughout different areas of life.

**Methods:**

Across three independent studies (*N* = 2646) we examined, through a series of online surveys, whether perceived personal control in different life domains (i.e., control over close relationships, health, work, and finances) mediates the linkages of perceived financial and health threats with subjective wellbeing (i.e., life satisfaction and happiness), self-rated health status, and psychological distress.

**Results:**

Higher perceived financial threat was related to diminished subjective wellbeing via perceived personal control over health, work (Studies 1 and 3), and finances (Study 3). Moreover, increased perceived health threat was associated with lower subjective wellbeing via perceived personal control over close relationships (Study 2) and health (Studies 2 and 3).

**Conclusion:**

Overall, these findings suggest that distinct domains of perceived personal control may underlie the relationships between various sources of perceived threat (economic vs. health) and well-being outcomes.

## Introduction

On March 2020 the SARS-CoV-2 disease, generally known as COVID-19, was characterized as a pandemic. Thereafter, several consequences of that situation arose on financial and healthcare levels, to the point where the pandemic turned into a socioeconomic and health crisis. Not only did it have effects over macrosocial spheres, the complexities of such a multidimensional event also presented psychosocial repercussions, with contact with others becoming more difficult, fears around health and economic status arising, and social behavior experiencing relevant changes (Moya and Willis, 2020).

This recent event underscored the enduring consequences of previous crises, highlighting that subjective evaluations of personal financial and health vulnerabilities were critical for comprehending the psychological impact of these sources of threat on wellbeing during periods of uncertainty. To gain further understanding on the psychological experience of financial and health threat, the present research aims to analyze their linkage to subjective wellbeing, self-rated health, and psychological distress. Nevertheless, this multistudy investigation is not only aimed at analyzing such connections. It also sets out to determine a possible pathway through which these forms of psychological threat are linked to wellbeing outcomes: perceived personal control. More specifically, and considering that sense of control can vary throughout diverse life areas (Casto, 2010; Lachman and Weaver, 1998a), this paper will examine the mediating role of perceived personal control over several life domains (i.e., close relationships, health, work, and finances) in the relationships of perceived financial and health threats with subjective wellbeing (i.e., satisfaction with life and happiness), self-rated health status, and psychological distress.

A recurring aspect of all financial crises is the global rise of financial instability. This is the context where the psychological experience of financial threat acquires relevance as a variable that refers to feelings of fear, uncertainty, and worry toward the stability and security of the economy of individuals (Marjanovic et al., 2013). This form of perceived threat is activated by situations where personal financial situations might be at risk (e.g., work instability, rise in personal debt or current situation of financial crisis). Nevertheless, the COVID-19 context of instability was different from others in that there was an added factor: the health crisis derived from the expansion of the pandemic, with health consequences such as the hundreds of millions of citizens infected by the virus (WHO, 2023) and the resulting heightened perceptions of health threat (Serrano-Montilla et al., 2021).

Numerous research studies have suggested that the subjective experience of financial threat during times of crisis or financial instability has a detrimental psychological impact. For instance, during the last stages of the Great Recession in Spain, it became apparent that individuals who experienced higher levels of perceived financial threat related to this crisis reported lower levels of satisfaction with life, happiness, and self-rated health status (Navarro-Carrillo et al., 2021). Furthermore, during periods of economic and health instability, individuals frequently encounter difficulties in estimating future outcomes since these are rare occurrences they have not experienced before. Thus, vulnerability to feelings such as uncertainty, threat, and anxiety increases. These unpredictable contexts could lower perceived personal control, understood as one's ability to affect important aspects of the environment (Fritsche et al., 2017). As a matter of fact, several investigations suggest that perceptions of financial threat during contexts of crisis are connected to lower levels of perceived personal control. For instance, Fritsche et al. (2017) found that participants who experience heightened perceived financial threat showed diminished levels of perceived personal control. Although research on perceived health threat might not be as extensive, evidence collected during the COVID-19 pandemic highlights how the perception of a threat as less dangerous was connected to lower levels of distress (Karademas and Thomadakis, 2023). Moreover, prolongued exposure to information pertaining this pandemic was connected to higher levels of depressive symptoms (Wu et al., 2024).

Available empiric research shows that diminished perceived personal control may have a negative impact on wellbeing and health. Some examples may be the presentation of lower levels of happiness and mental health (Han et al., 2023; Li et al., 2023) and poorer physical health outcomes (Hong et al., 2021). Regarding COVID-19, perceived personal control was determined to be able to prevent some of the negative effects perceived health threat could inflict over the population's life satisfaction (Zheng et al., 2020). However, it is important to note that most studies that have explored the connection between perceived personal control and indicators of wellbeing and mental health have considered perceived personal control as a unitary variable. In other words, it has traditionally been assessed through the global perception of perceived personal control. Nonetheless, some research suggests that levels of perceived personal control may substantially vary depending on the life domain that is being considered. In this regard, for instance, Lachman and Weaver (1998a) found that the relevance of different life domains changes throughout life, with perceived personal control over work increasing with age and perceived personal control over relationships with children getting lower over time. These results provide support to the idea that perceived personal control could be related to various aspects of life aspects and that differential levels of perceived personal control are expected to be found depending on the life domain that is being assessed.

Drawing on this approach, in this research we focused on four perceived personal control specific life domains: close relationships, health, work and finances. These are four areas that are linked to certain dimensions of life on which perceived personal control seems to be especially vulnerable (Casto, 2010; Conway et al., 1983; Lachman and Weaver, 1998a). Furthermore, these life domains take on significant relevance when considering perceptions of financial and health threats. Some studies have highlighted how situations that pose a danger to health rise prosocial tendencies (Serrano-Montilla et al., 2021) and closeness with loved ones (Li et al., 2020). Another aspect that is affected by unstable situations is work because of higher unemployment rates, inflation, and cutbacks. The work domain has been proven to be affected by changes in perceived personal control, with workers that could choose their work hours being less affected by the effects of demanding work schedules (Tucker et al., 2015). Moreover, having more work perceived personal control allows workers to reduce conflict and rise their wellbeing (Wood et al., 2018). Also, there is evidence that crises threaten individual's economic status, which arises worry toward their future ability to economically sustain themselves and, thus, lowers their perceived personal control (Fritsche et al., 2017).

Taking all of the above into consideration, the aim of this multi-study investigation was 2fold: (a) to test the contribution of perceived financial and health threats to various wellbeing and health outcomes; and (b) to determine which perceived personal control domains drive the perceived financial and health threats-wellbeing/health relationships. Study 1 focused on analyzing the potential mediating role of perceived personal control in different life domains in the relationships of perceived financial threat with subjective wellbeing and self-rated health status. We hypothesized that higher (1a) levels of perceived financial threat would be indicative of diminished subjective wellbeing and self-rated health status and that (2a) perceived personal control over work and health would mediate such associations. Study 2 expanded the previous study by incorporating perceived health threat and scrutinizing how this source of psychological threat was linked to subjective wellbeing and self-rated health status, while also controlling for perceived financial threat. Moreover, the potential mediating role of perceived personal control in different life domains in these relationships was also tested. We hypothesized that (1b) increased perceived health threat would be related to lower levels of subjective wellbeing and self-rated health status and that, in this case, (2b) perceived personal control over close relationships and health would mediate the perceived health threat-wellbeing outcomes linkages. Finally, pre-registered Study 3 aimed to replicate the findings of Studies 1 and 2 on a wide and diverse Spanish community sample. To broaden the nature of the indicators of wellbeing evaluated, psychological distress was also included. The hypotheses for this study were in line with the previous ones, expecting to find that (1c) perceived financial and health threats would be associated with lower levels of subjective wellbeing and self-rated health status, and with higher levels of psychological distress. We also predicted that (2c) perceived personal control over work and finances would mediate the connections between perceived financial threat and wellbeing outcomes and that (2d) perceived personal control over health and close relationships would mediate the perceived health threat and wellbeing outcomes linkages.

## Study 1

### Method

#### Participants

The sample consisted of 382 Spanish-speaking adults [138 men (36.1%), 231 women (60.5%), 9 participants who didn't want to say it (2.4%) and 4 who didn't identify as men or women (1%)] with ages ranging from 18 and 70 years old (*M* = 41.39, *SD* = 12.63). Supplementary Table S1 (found in Supplementary material) presents a description of the participants' sociodemographic characteristics.

Power analyses were carried out by using the G^*^Power 3.1 statistical software package (Faul et al., 2009). Our sample size (*N* = 1541) allowed us to detect correlation effects of ρ ≥ 0.14 (two-tailed) and regressions effects of *f* ^2^ ≥ 0.02 including eight predictors (i.e., gender, age, marital status, income, educational attainment, occupation, subjective socioeconomic status, and perceived financial threat) with power > 0.80 at α = 0.05.

#### Measures

##### Satisfaction with life

It was assessed by administering the well-validated Spanish version (Cabañero et al., 2004) of the Satisfaction with Life Scale (SWLS; Diener et al., 1985). It consists of 5 items (e.g., “If I could live my life over, I would change almost nothing”) presented in a 5-point Likert-type response format ranging from 1 (*strongly disagree*) to 5 (*strongly agree*). The scale has been proven to have a stable one factor structure, good internal consistency, and correspondence with other indicators of subjective wellbeing (Cabañero et al., 2004; Navarro-Carrillo et al., 2021).

##### Happiness

We used the Subjective Happiness Scale [SHS; Lyubomirsky and Lepper, 1999; Spanish version by Extremera and Fernández-Berrocal (2014)]. This instrument comprises 4 items (e.g., “Compared to most of my peers, I consider myself...”) on a 7-point Likert scale with scores ranging from 1 (e.g., *less happy*) to 7 (e.g., *happier*). Previous research has shown this scale has a one factor structure and an acceptable internal consistency, alongside correspondence with other indicators of subjective wellbeing (Extremera and Fernández-Berrocal, 2014; Navarro-Carrillo et al., 2018).

##### Self-rated health status

A one-item measure of self-perceived general health was used: “In general, would you say your health is…?”. It employs a 5-point Likert response scale (1= *very bad*; to 5 = *very good*). This measurement has shown consistent correspondence with other wellbeing indicators (Navarro-Carrillo et al., 2021).

##### Perceived financial threat

A Spanish-language version of the Financial Threat Scale (Marjanovic et al., 2013, 2015) was used. It comprises 5 items (e.g., “To which extent do you feel like you are at risk?”) with a 5-point Likert scale response format where 1 is *not at all* and 5 is *extremely*. The scale has shown very good reliability values and consistent correspondence with external variables relevant on a theoretical level (Navarro-Carrillo et al., 2021).

##### Personal control in different life domains

Perceived personal control was measured considering different life domains: close relationships, health, work, and finances. The wording of the questions was based on Bishop's control question (Bishop, 2005): “How much control do you have over your [life domain]?”. This resulted in 4 items where the participants were asked how much control they believed to have over each life domain (e.g., “How much control do you have over your close relationships?”). The response format was a 5-point Likert scale (e.g., 1 = *no control at all*; 5 = *absolute control*).

##### Objective social class

As in previous research (Navarro-Carrillo et al., 2020), objective social class was evaluated considering participants' income (1 = < 650€; 8 = >5,800€), educational attainment (1 = primary school; 8 = doctorate), and occupation [1 = technical professional occupations (e.g., doctor, teacher, engineer); 8 = unemployed].

##### Subjective socioeconomic status

We used the MacArthur Scale of Subjective Social Status (Adler et al., 2000) to assess participants perceived social class. This measure takes the form of a (social) ladder composed of 10 rungs representing different positions in the social hierarchy considering income, education, and occupation. We assigned a number from 1 to 10 to each rung (higher numbers reflected a higher position in the social class hierarchy). Participants were asked to select the number of the rung where they considered themselves to be in comparison with the rest of society.

#### Procedure

The sample was recruited by undergraduate students who distributed the online survey among their friends and family (i.e., snowball sampling), receiving partial academic credit in exchange for their involvement. Such online survey was located on the platform *Qualtrics*. Individuals were provided with a brief description of the study, had their anonymity and confidentiality ensured, were informed that they could stop answering at any given point if they decided to do so, and were asked for their consent to participate, after which they filled the survey. This study was carried out in accordance with the responsible university's ethical guidelines (report n° 1856/CEIH/2020) and the Declaration of Helsinki.

#### Data analyses

We calculated mean scores, standard deviations, and the patterns of bivariate correlations of all relevant variables, which can be found on Supplementary Table S3 alongside the Cronbach's Alpha values of the measures used (available in Supplementary material). To test whether perceived financial threat contributed to the prediction of subjective wellbeing and self-rated health status, several hierarchical regression analyses were conducted [specifically one for each indicator of subjective wellbeing (i.e., satisfaction with life and happiness) and self-rated health status as criterion variables]. In the first step of the regression equations, gender (1 = man, 2 = woman), age, and marital status (1 = single, 2 = in a relationship) were incorporated. In the second step, objective social class indicators (i.e., income, educational attainment, and occupation) and the subjective socioeconomic status measure were included. Perceived financial threat was added in the last step. Finally, to verify whether perceived personal control in different life domains mediated the effects of perceived financial threat on subjective wellbeing and self-rated health status, we conducted a set of parallel mediation analyses (one per subjective wellbeing indicator and one for self-rated health status as criterion variables) with 10.000 bootstraps, using model 4 of Hayes' PROCESS Macro for SPSS (Hayes, 2013). To supplement these analyses, we performed pairwise contrasts of indirect effects.

### Results

The database used for this study can be found at the Open Science Framework (OSF) repository: https://osf.io/jex6t/.

#### Descriptive statistics and bivariate correlations

Supplementary Table S1 presents the detailed frequencies of the sociodemographic characteristics of the sample. Supplementary Table S3 shows the descriptive statistics of the scales, their pattern of correlations, and the Cronbach's alpha of the survey measures.

#### Hierarchical regression analyses: the contribution of perceived financial threat to subjective wellbeing and self-rated health status

To test Hypothesis 1a, we performed three regression analyses (one per indicator of wellbeing/health). A detailed view of all three steps of the regression analyses is provided in Table 1.

**Table 1:** Hierarchical regression analyses predicting satisfaction with life, happiness, and self-rated health status from Study 1.

**Predictor**	**Satisfaction with life**			**Happiness**			**Self-rated health status**		
	β	***t***	Δ***R**^2^*	β	***t***	Δ***R**^2^*	β	***t***	Δ***R**^2^*
**Step 1**			0.06^**^			0.01			0.03^*^
Gender	−0.01	−0.27		0.04	0.80		−0.05	−0.91	
Age	0.06	1.06		0.07	1.22		−0.16^**^	−0.31	
Marital Status	0.22^**^	4.34		0.07	1.41		0.03	0.61	
**Step 2**			0.27^**^			0.13^**^			0.04^**^
Gender	0.01	0.11		0.06	1.09		−0.04	−0.71	
Age	−0.01	−0.12		0.00	0.07		−0.20^**^	−3.56	
Marital Status	0.16^**^	3.6		0.05	1.04		0.01	0.13	
Income	0.05	0.92		−0.03	−0.49		0.02	0.29	
Educational attainment	0.05	0.96		−0.16^**^	−2.82		−0.04	−0.63	
Occupation	−0.02	−0.35		−0.02	−0.41		−0.10	−1.69	
SES	0.48^**^	9.87		0.38^**^	6.97		0.15^*^	2.57	
**Step 3**			0.07^**^			0.14^**^			0.02^**^
Gender	0.01	0.30		0.07	1.42		−0.03	−0.64	
Age	−0.01	−0.19		0.00	−0.01		−0.20^**^	−3.62	
Marital Status	0.12^**^	2.74		−0.01	−0.18		−0.02	−0.32	
Income	0.03	0.60		−0.05	−1.0		0.01	0.14	
Educational attainment	0.07	1.43		−0.13^**^	−2.54		−0.03	−0.45	
Occupation	0.01	0.15		0.01	0.21		−0.09	−1.48	
SES	0.38^**^	7.9		0.25^**^	4.68		0.10	1.61	
Financial threat	−0.30^**^	−6.63		−0.41^**^	−8.25		−0.16^**^	−2.80	

^*^ p < 0.05, ^**^ p < 0.01. SES, Subjective socioeconomic status.

Regarding satisfaction with life, the results showed that those participants that were in a relationship (β = 0.12, *p* = 0.006) and those who reported higher subjective socioeconomic status levels (β = 0.38, *p* < 0.001) were more satisfied with their lives. Consistent with our expectations, participants with higher scores on perceived financial threat were less satisfied with their lives (β = −0.30, *p* < 0.001). The addition of perceived financial threat in the third step accounted for a significant variance increment in the criterion variable of 7.4%, Δ*F*_(1, 360)_ = 43.97, *p* < 0.001.

With regard to happiness, participants who considered that they belonged to higher social class levels reported higher levels of happiness (β = 0.25, *p* < 0.001). In line with our expectations, participants with higher scores of perceived financial threat showed lower levels of happiness (β = −0.41, *p* < 0.001). Adding perceived financial threat in step 3 accounted for a significant increment in the criterion variable's variance of 13.7%, Δ*F*_(1, 360)_ = 67.98, *p* > 0.001.

Concerning self-rated health status, the results indicated that the older the participants were, the worse their self-rated health status was (β = −0.20, *p* < 0.001). Moreover, those who were more financially threatened also perceived that their health was worse (β = −0.16, *p* = 0.005). Adding perceived financial threat to the third step of the regression equation accounted for a criterion variable variance increase of 2%, Δ*F*_(1, 360)_ = 7.82, *p* = 0.005.

Importantly, the associations between perceived financial threat and wellbeing and health emerged beyond the variance accounted for by sociodemographic factors and objective and subjective socioeconomic status indexes.

#### Testing the mediating role of perceived personal control in different life domains

To test Hypothesis 2a, several parallel mediation analyses were run, one per indicator of wellbeing.

**Figure 1:**
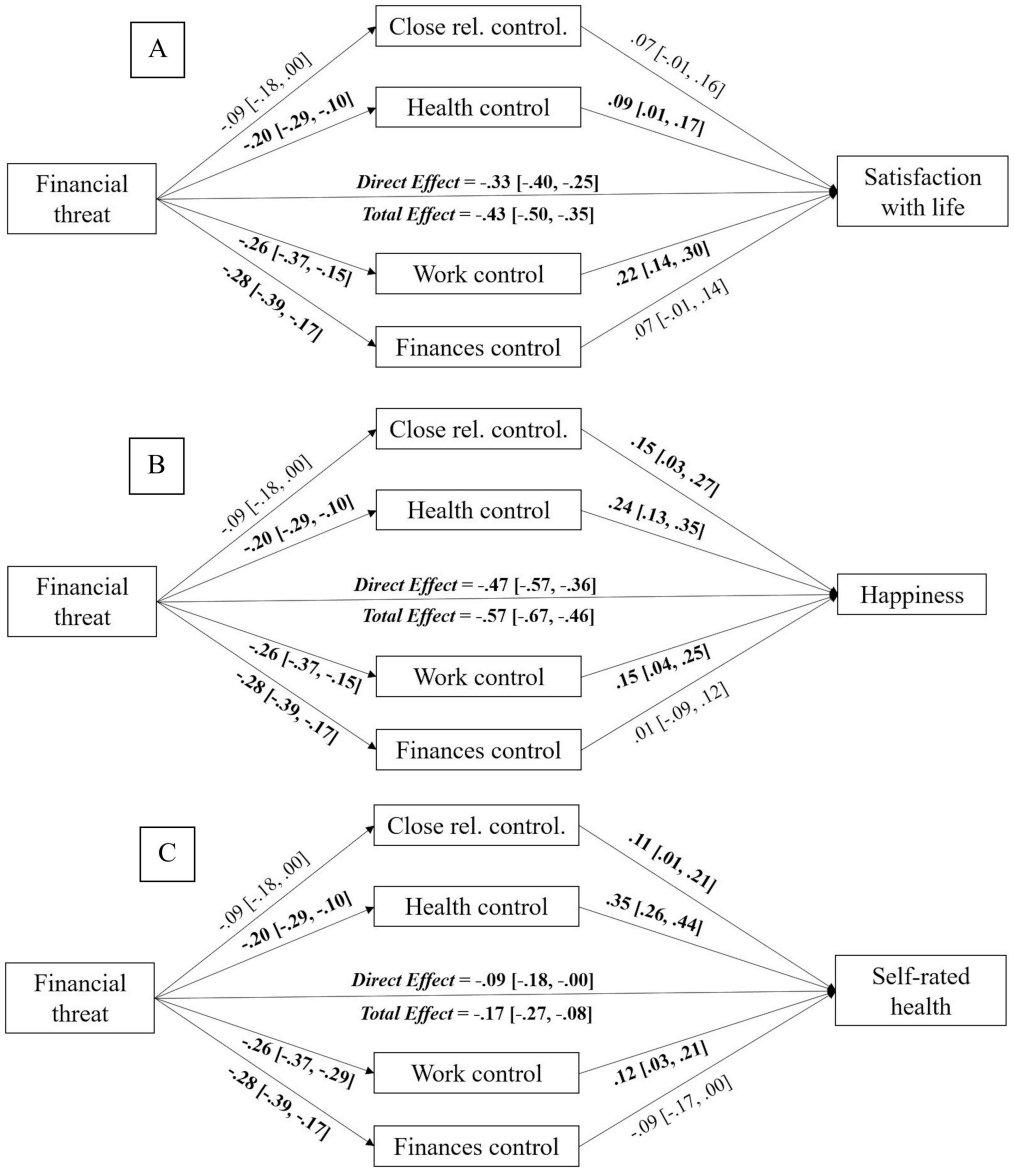
Mediation models displaying the indirect effects of perceived financial threat on satisfaction with life **(A)**, happiness **(B)**, and self-rated health status **(C)** via perceived personal control in different life domains. Unstandardized estimates, with 95% CI reported between brackets. Significant effects appear in bold text. The bootstrap sample size was 10,000. Close rel., close relationships.

In regard to satisfaction with life, our results showed that perceived financial threat was indirectly related to satisfaction with life via perceived personal control over health [*b* = −0.02, *SE* = 0.01, 95% CI (−0.04, −0.00)] and work [*b* = −0.06, *SE* = 0.02, 95% CI (−0.09, −0.03)]. Perceived personal control over close relationships [*b* = −0.01, *SE* = 0.01, 95% CI (−0.02, 0.00)] and over finances [*b* = −0.02, *SE* = 0.01, 95% CI (−0.05, 0.00)] did not emerge as mediating factors. The direct effect of perceived financial threat on satisfaction with life [*b* = −0.33, *SE* = 0.04, 95% CI (−0.40, −0.25)] remaining significant after the inclusion of the mediating variables (i.e., perceived personal control in different life domains), indicating the existence of partial mediations. The coefficients and confidence intervals of all paths are presented in Figure 1A.

Concerning happiness, perceived personal control over health [*b* = −0.05, *SE* = 0.02, 95% CI (−0.08, −0.02)] and work [*b* = −0.04, *SE* = 0.02, 95% CI (−0.08, −0.01)] mediated the association of perceived financial threat with happiness. However, perceived personal control over close relationships [*b* = −0.01, *SE* = 0.01, 95% CI (−0.04, 0.00)] and finances [*b* = −0.00, *SE* = 0.02, 95% CI (−0.03, 0.03)] did not mediate that relationship. As in the preceding case, the direct effect of perceived financial threat on happiness [*b* = −0.47, *SE* = 0.05, 95% CI (−0.57, −0.36)] remained significant after including the mediating variables (i.e., perceived personal control in different life domains), indicating again the existence of partial mediations. The remaining paths are represented in Figure 1B.

In the case of self-rated health status, parallel mediation analyses also showed that the relationship between perceived financial threat and self-rated health status was mediated by perceived personal control over health [*b* = −0.07, *SE* = 0.02, 95% CI (−0.11, −0.03)] and work [*b* = −0.03, *SE* = 0.01, 95% CI (−0.06, −0.01)]. Perceived personal control over close relationships [*b* = −0.01, *SE* = 0.01, 95% CI (−0.03, 0.00)] and finances [*b* = 0.02, *SE* = 0.01, 95% CI (−0.00, 0.05)] did not show significant indirect effects. Since the direct effect of perceived financial threat over self-rated health [*b* = −0.09, *SE* = 0.04, 95% CI (−0.18, −0.00)] remained significant after the inclusion of the mediating factors (i.e., perceived personal control in different life domains), the mediation was partial. As in the previous analyses, paths not reported in the text are presented in Figure 1C.

To sum up, our results showed that greater perceived financial threat was indirectly related to reduced satisfaction with life, happiness, and self-rated health status via perceived personal control over health and work life spheres.

## Study 2

### Method

#### Participants

The sample comprised a total of 723 Spanish-speaking adults of the general population [198 men (27.4%), 512 women (70.8%), 7 (1%) individuals who did not want to specify it and 6 (0.8%) who did not identify as men or women]. Their ages ranged from 18 to 65 years old (*M* = 23.54, *SD* = 5.78). Supplementary Table S1 presents the participant's sociodemographic characteristics.

Power analyses were performed by utilizing G^*^Power 3.1 statistical software package (Faul et al., 2009). The current sample size (*N* = 723) allowed us to detect correlation effects of ρ ≥ 0.10 (two-tailed) and regressions effects of *f*^2^ ≥ 0.01 including nine predictors (i.e., gender, age, marital status, income, educational attainment, occupation, subjective socioeconomic status, perceived financial threat, and perceived health threat) with power >0.80 at α = 0.05.

#### Instruments

*Satisfaction with life, happiness, self-rated health status, and perceived financial threat*. We employed the same measures used in Study 1.

##### Perceived health threat

The Financial Threat Scale (Marjanovic et al., 2013) was adapted to the health context (i.e., “Please, indicate how you feel in regards to your health, answering the questions bellow”), maintaining the 5-item structure and the 5-point Likert scale response format from the original version. Other previous studies, such as the one carried out by Serrano-Montilla et al. (2021), already satisfactorily adapted this measure to the health field.

##### Perceived personal control in different life domains

Each of the four separate control domains were measured the same way as in Study 1.

##### Objective and subjective socioeconomic status

These factors of social class were evaluated as they were in Study 1. The descriptive statistics of these measures can be observed in Supplementary Table S1.

#### Procedure

The link to the online survey located on the platform *Qualtrics* was spread through the local University's email channels that reached all students, professors and personnel (i.e., incidental sampling). Individuals were provided a brief description of the study; their anonymity and confidentiality were ensured, and were informed that they could stop answering at any given point if they decided to do so. Afterwards, they gave their consent to participate and filled the survey. Participants could enter a drawing for a 50-euro prize after completing the survey. This study was carried out in accordance with the responsible university's ethical guidelines (report n° 1856/CEIH/2020) and the Declaration of Helsinki.

#### Data analyses

Mean scores and standard deviations were computed for all research variables, which can be found in Supplementary Table S4. We also calculated the internal consistency of the measures using Cronbach's alpha (α) coefficient. Moreover, to test whether perceived health threat contributed to the prediction of subjective wellbeing and self-rated health status, we performed a set of hierarchical regression analyses with satisfaction with life, happiness, and self-rated health status as criterion variables. In the first step of the regression equations, we incorporated gender (1 = man, 2 = woman), age, and marital status (1 = single, 2 = in a relationship). In the second step, we included objective social class indicators (i.e., income, educational attainment, and occupation) and the subjective socioeconomic status measure. Finally, perceived financial and health threats were added in the last step. Afterwards, in order to verify whether perceived personal control in different life domains mediated the effects of perceived health threat on subjective wellbeing and self-rated health status, a set of parallel mediation analyses (one per subjective wellbeing indicator and one for self-rated health status as criterion variables, with perceived financial threat as covariate) were conducted. We used model 4 of Hayes' PROCESS Macro for SPS with 10.000 bootstraps (Hayes, 2013). To supplement these analyses, we performed pairwise contrasts of indirect effects.

### Results

The database used for this study can be found at OSF: https://osf.io/jex6t/.

#### Descriptive statistics and bivariate correlations

Supplementary Table S1 presents the detailed frequencies of the sociodemographic characteristics of the sample. Supplementary Table S4 displays the descriptive statistics of the measures included in this study and their bivariate correlations.

#### Hierarchical regression analyses: the contribution of perceived health threat to subjective wellbeing and self-rated health status

To test Hypothesis 1b, three hierarchical regression analyses were performed. A more detailed view of all three steps of the regression analyses can be found in Table 2.

**Table 2:** Hierarchical regression analyses predicting satisfaction with life, happiness, and self-rated health status from Study 2.

**Predictor**	**Satisfaction with life**			**Happiness**			**Self-rated health status**		
	β	***t***	Δ***R**^2^*	β	***t***	Δ***R**^2^*	β	***t***	Δ***R**^2^*
**Step 1**			0.02^**^			0.01^*^			0.01
Gender	0.10^**^	2.76		−0.02	−0.49		−0.08^*^	−2.03	
Age	−0.07	−1.90		0.06	1.63		−0.02	−0.44	
Marital Status	0.09	2.28		0.07	1.93		0.04	1.06	
**Step 2**			0.20^**^			0.14^**^			0.08^**^
Gender	0.09^**^	2.61		−0.03	−0.78		−0.08^*^	−2.21	
Age	−0.11^**^	−3.13		0.03	0.79		−0.04	−0.10	
Marital Status	0.09^**^	2.65		0.08^*^	2.11		0.04	1.18	
Income	−0.01	−0.37		−0.00	−0.11		0.10^*^	2.52	
Educational attainment	0.09^*^	2.51		0.06	1.70		0.08^*^	2.06	
Occupation	−0.07^*^	−2.08		−0.08^*^	−2.23		−0.05	−1.38	
SES	0.41^**^	11.14		0.33^**^	8.66		0.19^**^	4.86	
**Step 3**			0.09^**^			0.14^**^			0.10^**^
Gender	0.10^**^	3.26		−0.01	−0.22		−0.06	−1.74	
Age	−0.12^**^	−3.56		0.02	0.55		−0.05	−1.40	
Marital Status	0.09^**^	2.79		0.08^*^	2.30		0.05	1.35	
Income	−0.05	−1.52		−0.05	−1.50		0.07	1.86	
Educational attainment	0.08^*^	2.41		0.05	1.53		0.07	1.86	
Occupation	−0.07^*^	−1.98		−0.07^*^	−2.17		−0.05	−1.40	
SES	0.33^**^	9.03		0.24	6.38		0.16^**^	4.16	
Financial threat	−0.14^**^	−3.23		−0.16^**^	−3.56		0.10^*^	2.03	
Health threat	−0.20^**^	−4.72		−0.27^**^	−6.22		−0.38^**^	−8.26	

^*^ p < 0.05, ^**^ p < 0.01. SCC, Subjective socioeconomic status.

Considering satisfaction with life, the results showed that women (β = 0.10, *p* = 0.001), younger participants (β = −0.12, *p* < 0.001), those in a relationship (β = 0.09, *p* = 0.005), those with higher educational attainment (β = 0.08, *p* = 0.02), those with higher paying jobs (β = −0.07, *p* = 0.05) and those who considered that they belonged to a higher social class (β = 0.33, *p* < 0.001) were more satisfied with their lives. Participants with higher scores of perceived financial threat were less satisfied with their lives (β = −0.14, *p* = 0.001). Finally, consistent with our expectations, participants with higher levels of perceived health threat were less satisfied with their lives (β = −0.20, *p* < 0.001). The addition of perceived financial and health threats in the third step of the regression equation accounted for a significant variance increment of 8.7%, Δ*F*_(2, 700)_ = 44.18, *p* < 0.001, in the criterion variable.

With regard to happiness, participants who were in a relationship (β = 0.08, *p* = 0.02), had a higher paying job (β = −0.07, *p* = 0.03) and belonged to a higher social class (β = 0.24, *p* < 0.001) were happier. Participants presenting higher levels of perceived financial threat were less happy (β = −0.16, *p* < 0.001). Confirming our Hypothesis 2b, higher levels of perceived health threat were predictive of lower levels of happiness (β = −0.27, *p* < 0.001). The inclusion of the psychological experiences of threat in the regression equations resulted in a significant variance increment in the criterion variable of 13.8%, Δ*F*_(2, 700)_ = 67.35, *p* < 0.001.

In reference to self-rated health status, we found that those who considered that they belonged to a higher social class (β = 0.16, *p* < 0.001) had better health. The perception of higher health threat was associated with a lower self-rated health status (β = −0.38, *p* < 0.001). An increase of 10%, ΔF_(2, 700)_ = 43.01, *p* < 0.001, on explained variance of the criterion variable was obtained in the last step of the regression.

Notably, the effects of perceived financial and health threats detailed above were sustained even after accounting for multiple sociodemographic and social class aspects.

#### Testing the mediating role of perceived personal control in different life domains

**Figure 2:**
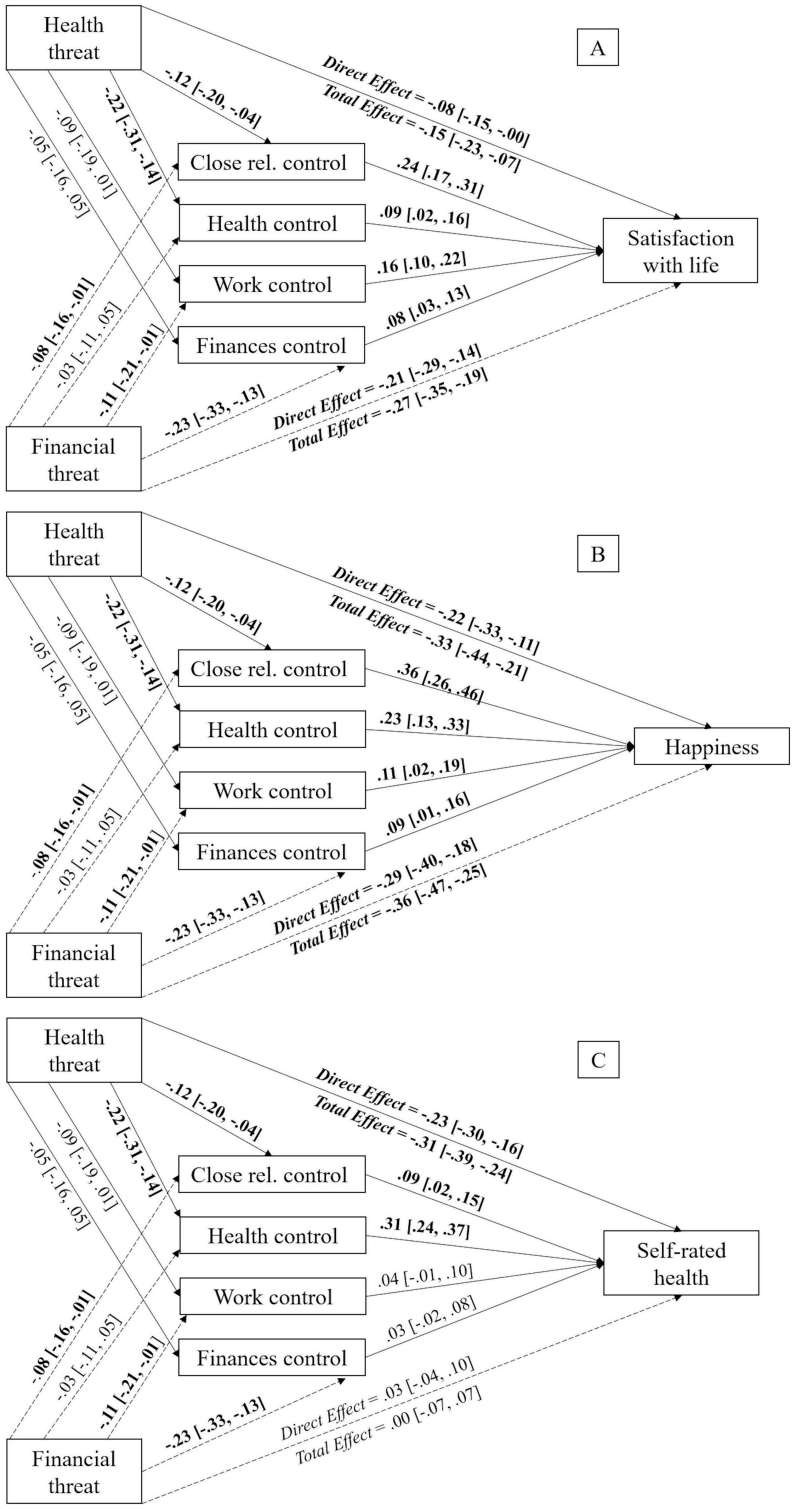
Mediation models displaying the indirect effects of perceived health threat on satisfaction with life **(A)**, happiness **(B)**, and self-rated health status **(C)** via perceived personal control in different life domains with perceived financial threat as covariate. Unstandardized estimates, with 95% CI reported between brackets. Significant effects appear in bold text. The bootstrap sample size was 10,000. Close rel., close relationships.

To confirm Hypothesis 2b, three parallel mediation analyses were run. In the case of satisfaction with life, perceived personal control over close relationships [*b* = −0.03, *SE* = 0.01, 95% CI (−0.05, −0.01)] and over health [*b* = −0.02, *SE* = 0.01, 95% CI (−0.04, −0.00)] had a mediating effect on the relationship between perceived health threat and life satisfaction. However, perceived personal control over work [*b* = −0.01, *SE* = 0.01, 95% CI (−0.03, 0.00)] and over finances [*b* = −0.00, *SE* = 0.01, 95% CI (−0.02, 0.00)] did not emerge as mediating factors. The direct effect of perceived health threat over satisfaction with life [*b* = −0.08, *SE* = 0.04, 95% CI (−0.15, −0.00)] remained significant after the inclusion of the mediating variables (i.e., perceived personal control over health), indicating the existence of partial mediations, as shown in Figure 2A.

Focusing on happiness, it was found that perceived personal control over close relationships [*b* = −0.04, *SE* = 0.02, 95% CI (−0.08, −0.01)] and over health [*b* = −0.05, *SE* = 0.02, 95% CI (−0.08, −0.02)] and had a mediating effect on the relationship between perceived health threat and happiness. However, perceived personal control over work [*b* = −0.01, *SE* = 0.01, 95% CI (−0.03, 0.00)] and over finances [*b* = −0.00, *SE* = 0.01, 95% CI (−0.02, 0.01)] did not show mediating effects. The direct effect of perceived health threat over happiness [*b* = −0.22, *SE* = 0.06, 95% CI (−0.33, −0.11)] remained significant after the inclusion of the mediating variables (i.e., perceived personal control over close relationships), meaning these were partial mediations, as shown in Figure 2B.

Finally, regarding self-rated health status, perceived personal control over close relationships [*b* = −0.01, *SE* = 0.01, 95% CI (−0.02, −0.00)] and over health [*b* = −0.07, *SE* = 0.02, 95% CI (−0.10, −0.04)] mediated the relationship between perceived health threat and self-rated health status. Nevertheless, perceived personal control over work [*b* = −0.00, *SE* = 0.00, 95% CI (−0.01, 0.00)] and over finances [*b* = −0.00, *SE* = 0.00, 95% CI (−0.01, 0.00)] did not emerge as mediating variables. Mediations were partial due to the direct effect of perceived health threat on self-rated health status [*b* = −0.23, *SE* = 0.04, 95% CI (−0.30, −0.16)] remaining significant after the inclusion of the different mediating factors (i.e., perceived personal control over health), which can be seen in Figure 2C.

To sum up, our results showed that greater perceived health threat was indirectly related to reduced satisfaction with life, happiness, and self-rated health status via perceived personal control over close relationships and health life spheres.

## Study 3

### Method

#### Participants

A total of 1,541 adults of the Spanish general population [759 men (49.3%), 779 women (50.7%) and 3 “other” (0.3%)], with ages ranging from 18 to 95 years old (*M* = 50.99, *SD* = 18.42) participated in this study. Supplementary Table S2 presents the participant's sociodemographic characteristics.

Power analyses were carried out by using the G^*^Power 3.1 statistical software package (Faul et al., 2009). Our sample size (*N* = 382) allowed us to detect correlation effects of ρ ≥ 0.07 (two-tailed) and regressions effects of *f*^2^ ≥ 0.006 including 10 predictors (i.e., gender, age, marital status, income, educational attainment, subjective socioeconomic status regarding income, educational attainment and occupational status, perceived financial threat and perceived health threat) with power >0.80 at α = 0.05.

#### Instruments

##### Satisfaction with life and happiness

Satisfaction with life and happiness were assessed using the same measures as in Studies 1 and 2.

##### Self-rated health status

The self-rated health item (i.e., “How would you describe your health these days?”; Benyamini and Idler, 1999) was used. It presented a Likert scale from 1 (“*the worst health possible*”) to 10 (“*the best health possible*”).

##### Perceived financial threat

Two items adapted from Fritsche et al. (2017) were used. Participants were asked to rate their levels of worry about their personal economic situation when considering the situation caused by the COVID-19 pandemic in Spain from 1 (“*none*”) to 7 (“*a lot*”).

##### Perceived health threat

Perceived health threat was assessed adapting the two perceived financial threat items to the health context, asking participants about their levels of worry toward their health status.

##### Psychological distress

It was measured using the Spanish version of the PHQ-4 (Cano-Vindel et al., 2018; Kroenke et al., 2009). Respondents were presented with 4 issues (e.g., “Not being able to stop or control worrying”) and had to indicate how frequently they had experimented each of them in the last 2 weeks on a scale from 0 (*not at all*) to 3 (*almost daily*).

##### Perceived personal control in different life domains

Each of the four separate control domains were measured the same way as in Study 1 and 2.

##### Objective social class

Monthly income was evaluated by asking participants to write the approximate amount of money their household perceived each month. Afterwards those amounts were coded using the same ranges as in Studies 1 and 2 (1 = < 650€; 8 = > 5,800€). Educational attainment was measured asking participants to choose from a list the highest level of education they had achieved (1 = unfinished primary school; 8 = doctorate).

##### Subjective socioeconomic status

These indicators of social class were evaluated through an adaptation of the McArthur Scale of Subjective Social Status (Adler et al., 2000) developed by Navarro-Carrillo et al. (2020). This adaptation consists on the assessment of subjective social class considering components of income, educational level, and job status independently and separately. Thus, respondents were asked to indicate their social class in a scale from 1 (“low social class”) a 10 (“high social class”) three times (one for each of the previously mentioned indicators and in comparison to the rest of the members of society). This way of assessing subjective social class has been proven to have a higher predictive utility in regards to wellbeing in comparison to the conventional MacArthur's Scale of Subjective Social Status (Navarro-Carrillo et al., 2020).

#### Procedure

Participants were collected through the company NetQuest. Based on the stratification by quotas regarding social class, gender, age, and region of residence as established by the Nielsen standards, this company follows the distribution of the Spanish population published by the National Statistics Institute of Spain. The survey included three attention checks and considered acquiescent responses and irregular response patterns. Just as the previous studies, the present research was approved by the responsible university's ethical guidelines (report n° 1856/CEIH/2020) and was conducted in accordance with the Declaration of Helsinki. The preregistration of this study can be found at OSF: https://osf.io/ncw4s/.

#### Data analyses

We computed mean scores and standard deviations for all research variables, their pattern of correlations and the Cronbach's alpha (α) coefficient of all the measures used, all reported in Supplementary Table S5. Next, four hierarchical regression analyses were run, one per indicator of wellbeing, health, and psychological distress. The first step of the regression included, as in the two previous studies, gender (1 = man, 2 = woman), age, and marital status (1 = in a relationship, 2 = single). The second step added objective social class indicators (i.e., family income and educational attainment) and subjective socioeconomic status indicators (i.e., income, educational attainment, and work status). Finally, the last step included both perceived financial and health threats. Finally, eight sets of parallel mediations were performed in order to test whether perceived personal control in different life domains mediated the effects of perceived financial and health threats on subjective wellbeing, self-rated health status, and psychological distress. Model 4 of Hayes' PROCESS Macro for SPSS (Hayes, 2013) with 10.000 bootstraps was employed. When an indicator of perceived threat was being considered as a predictor the other was also accounted for in the analytical models. As in the previous studies, to supplement these analyses, we performed pairwise contrasts of indirect effects.

### Results

The database used for this study can be found at the Open Science Framework repository: https://osf.io/jex6t/.

#### Descriptive statistics and bivariate correlations

Supplementary Table S2 presents the detailed frequencies of the sociodemographic characteristics of the sample.

Supplementary Table S5 presents the descriptive statistics of the scales, their bivariate correlations, and the Cronbach's alpha for the questionnaire measures.

#### Hierarchical regression analyses: the contribution of perceived financial and health threats to subjective wellbeing and self-rated health status

To test Hypothesis 1c, four hierarchical regression analyses were performed. A more detailed view of all three steps of the regression analyses can be found in Table 3.

**Table 3:** Hierarchical regression analyses predicting satisfaction with life, happiness, self-rated health status, and psychological distress from Study 3.

**Predictor**	**Satisfaction with life**			**Happiness**			**Self-rated health status**			**Psychological distress**		
	β	***t***	Δ***R**^2^*	β	***t***	Δ***R**^2^*	β	***t***	Δ***R**^2^*	β	***t***	Δ***R**^2^*
**Step 1**			0.07^**^			0.06^*^			0.01^*^			0.16^**^
Gender	−0.01	−0.41		0.05	1.83		−0.01	−0.37		0.13^**^	4.94	
Age	0.11^**^	4.00		0.21^**^	7.43		0.03	1.12		−0.30^**^	−11.69	
Marital Status	−0.22^**^	−8.75		−0.14^*^	−5.64		−0.07^*^	−2.59		0.08^**^	3.55	
**Step 2**			0.14^**^			0.05^**^			0.06^**^			0.04^**^
Gender	0.03	1.08		0.07^*^	2.62		0.01	0.33		0.11^**^	4.06	
Age	0.08^*^	3.08		0.17^**^	6.39		0.00	0.13		−0.29^**^	−11.15	
Marital Status	−0.17^**^	−7.35		−0.12^**^	−4.96		−0.05	−1.92		0.06^*^	2.31	
Income	0.02	0.61		−0.02	−0.92		−0.04	−1.57		−0.04	−1.58	
Educational attainment	−0.01	−0.47		−0.08^*^	−2.69		−0.05	−1.66		0.01	0.22	
Income SES	0.22^**^	7.50		0.12^**^	3.96		0.15^**^	4.78		−0.09^*^	−2.90	
Educational attainment SES	−0.04	−1.30		0.03	0.78		0.01	0.42		0.05	1.51	
Work status SES	0.22^**^	7.00		0.15^**^	4.47		0.13^**^	3.91		−0.14^**^	−4.58	
**Step 3**			0.04^**^			0.03^**^			0.13^**^			0.08^**^
Gender	0.04	1.46		0.08^*^	2.97		0.02	0.93		0.09^**^	3.75	
Age	0.08^*^	2.97		0.20^**^	7.25		0.08^*^	3.01		−0.30^**^	−11.83	
Marital Status	−0.17^**^	−7.50		−0.12^**^	−5.06		−0.05^*^	−2.12		0.06^*^	2.44	
Income	−0.01	−0.23		−0.03	−1.12		−0.04	−1.46		−0.02	−0.72	
Educational attainment	−0.03	−1.20		−0.09^*^	−3.05		−0.06^*^	−2.09		0.03	1.11	
Income SES	0.19^**^	6.27		0.12^**^	3.77		0.18^**^	5.71		−0.05	−1.68	
Educational attainment SES	−0.03	−0.98		0.02	0.75		0.00	0.01		0.04	1.26	
Work status SES	0.18^**^	5.98		0.12^**^	3.86		0.10^*^	3.32		−0.10^*^	−3.29	
Financial threat	−0.16^**^	−5.67		−0.02	−0.51		0.11^**^	3.80		0.16^**^	5.76	
Health threat	−0.10^**^	−3.76		−0.17^**^	−6.32		−0.40^**^	−15.23		0.18^**^	7.32	

^*^ p < 0.05, ^**^ p < 0.01. SES, Subjective socioeconomic status.

In regards to satisfaction with life, the results showed that older participants (β = 0.08, *p* = 0.003), those in a relationship (β = −0.17, *p* < 0.001), those that considered they belonged to a higher income bracket (β = 0.19, *p* < 0.000), those with higher occupational status (β = 0.18, *p* < 0.001) were more satisfied with their lives. Moreover, those who scored higher levels of perceived financial threat (β = −0.16, *p* < 0.001) and higher levels of perceived health threat (β = −0.10, *p* < 0.001) showed lower levels of satisfaction with life. The addition of perceived financial and health threats to the final step of the regression analysis accounted for a criterion variable's variance increase of 4.1%, Δ*F*_(2, 1, 527)_ = 41.84, *p* < *0.0*01.

Regarding happiness, results also showed that women (β = 0.08, *p* = 0.003), older participants (β = 0.20, *p* < 0.001), those in a relationship (β = −0.12, *p* < 0.001), with lower educational attainment (β = −0.09, *p* = 0.002), that considered they belonged to a higher income bracket (β = 0.12, *p* < 0.001) and with higher occupational status (β = 0.12, *p* < 0.001) were happier. Also, participants who showed increased perceived health threat (β = −0.17, *p* < 0.001) were less happy. Perceived financial threat showed no predictive effect (β = −0.02, *p* = 0.612). The addition of perceived financial and health threats to the last step of the regression analysis brought forth an increase of the criterion variable's variance of a 3.1%, Δ*F*_(2, 1, 527)_ = 27.41, *p* < *0.0*01.

Concerning self-rated health status, we found that those who were older (β = 0.08, *p* = 0.003), that were in a relationship (β = −0.05, *p* = 0.035), that achieved lower educational levels (β = −0.06, *p* = 0.031), considered they belonged to a higher income bracket (β = 0.18, *p* < 0.001), with higher occupational status (β = 0.10, *p* = 0.001) considered their health to be better. Furthermore, participants who experienced higher levels of perceived health threat (β = −0.40, *p* < 0.001) showed lower levels of self-rated health status. Interestingly, participants who presented higher levels of perceived financial threat also presented higher levels of self-rated health (β = 0.11, *p* < 0.001). Including perceived financial and health threats in the final step of the regression equation resulted in an increase on the criterion variable's variance of 13%, Δ*F*_(2, 1, 527)_ = 122.49, *p* < *0.0*01.

With regard to psychological distress, women (β = 0.09; *p* < 0.001), participants who were younger (β = −0.30; *p* < 0.001), single (β = 0.06; *p* = 0.015), perceived that they had lower occupational status (β = −0.10; *p* = 0.001) and higher levels of perceived financial (β = 0.16; *p* < 0.001) and health (β = 0.18; *p* < 0.001) threat showed higher levels of psychological distress. There was an increase on explained variance of mental health in the last steps of the regression analysis of 7.6%, ΔF_(2, 1, 527)_ = 79.75, *p* < 0.001. Remarkably, the effects of perceived financial and health threats on wellbeing that were found were obtained after controlling for several sociodemographic factors.

#### Perceived financial threat, perceived personal control in different life domains, and wellbeing/psychological distress: testing the mediating role of perceived personal control in different life domains

To test Hypothesis 2c, four parallel mediation analyses were performed, one per indicator of subjective wellbeing, one for self-rated health status, and another one for psychological distress. Perceived health threat was included as a covariate so as to control for the possible effects it may have over these indicators of wellbeing.

**Figure 3:**
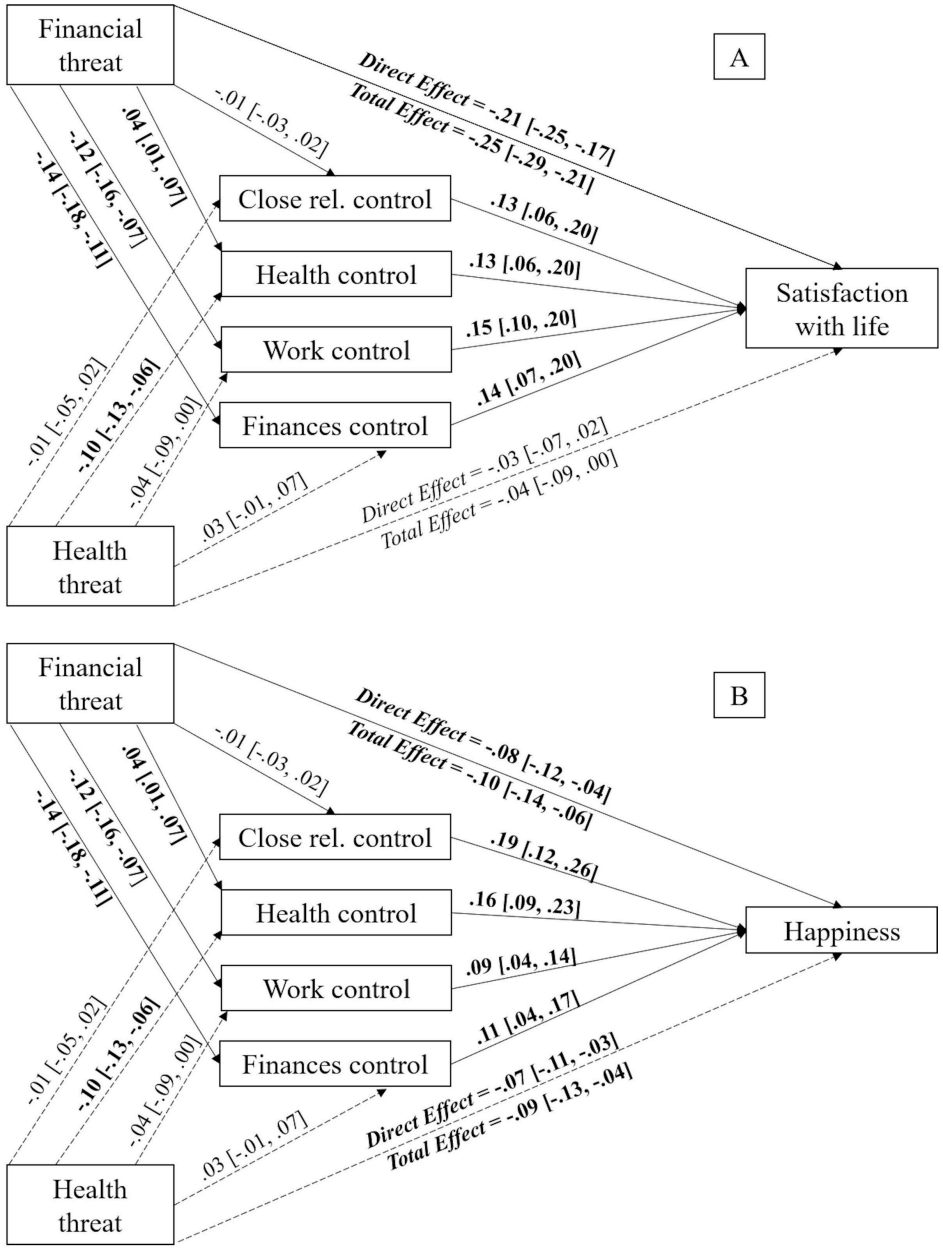
Mediation models displaying the indirect effects of perceived financial threat on satisfaction with life **(A)** and happiness **(B)** via perceived personal control in different life domains with perceived health threat as covariate. Unstandardized estimates, with 95% CI reported between brackets. Significant effects appear in bold text. The bootstrap sample size was 10,000. Close rel., close relationships.

When considering the connection between perceived financial threat and satisfaction with life, the results showed that perceived personal control over health [*b* = 0.01, *SE* = 0.00, 95% CI (0.00, 0.01)], work (*b* = −0.02, *SE* = 0.00, 95% CI (−0.03, −0.01)] and finances [*b* = −0.02, *SE* = 0.01, 95% CI (−0.03, −0.08)] mediated that relationship. However, perceived personal control over close relationships [*b* = −0.00, *SE* = 0.00, 95% CI (−0.01, 0.00)] did not. Since the direct effect of perceived financial threat over satisfaction with life [*b* = −0.21, *SE* = 0.02, 95% CI (−0.25, −0.17)] remained significant after the inclusion of the mediating variables (i.e., perceived personal control over health), these are considered partial mediations. The remaining paths can be observed in Figure 3A.

In regards to happiness, the previous results were replicated, finding that perceived personal control over health [*b* = 0.01, *SE* = 0.00, 95% CI (0.00, 0.01)], work [*b* = −0.01, *SE* = 0.00, 95% CI (−0.02, −0.00)] and finances [*b* = −0.02, *SE* = 0.01, 95% CI (−0.03, −0.01)] had a mediating effect on the relationship between perceived financial threat and happiness. However, perceived personal control over close relationships [*b* = −0.00, *SE* = 0.00, 95% CI (−0.01, 0.01)] did not present any mediating effect. The effect of perceived financial threat over happiness [*b* = −0.08, *SE* = 0.02, 95% CI (−0.12, −0.04)] remained significant after the inclusion of the mediating variables (i.e., perceived personal control over work), meaning this represents a partial mediation, as shown in Figure 3B.

**Figure 4:**
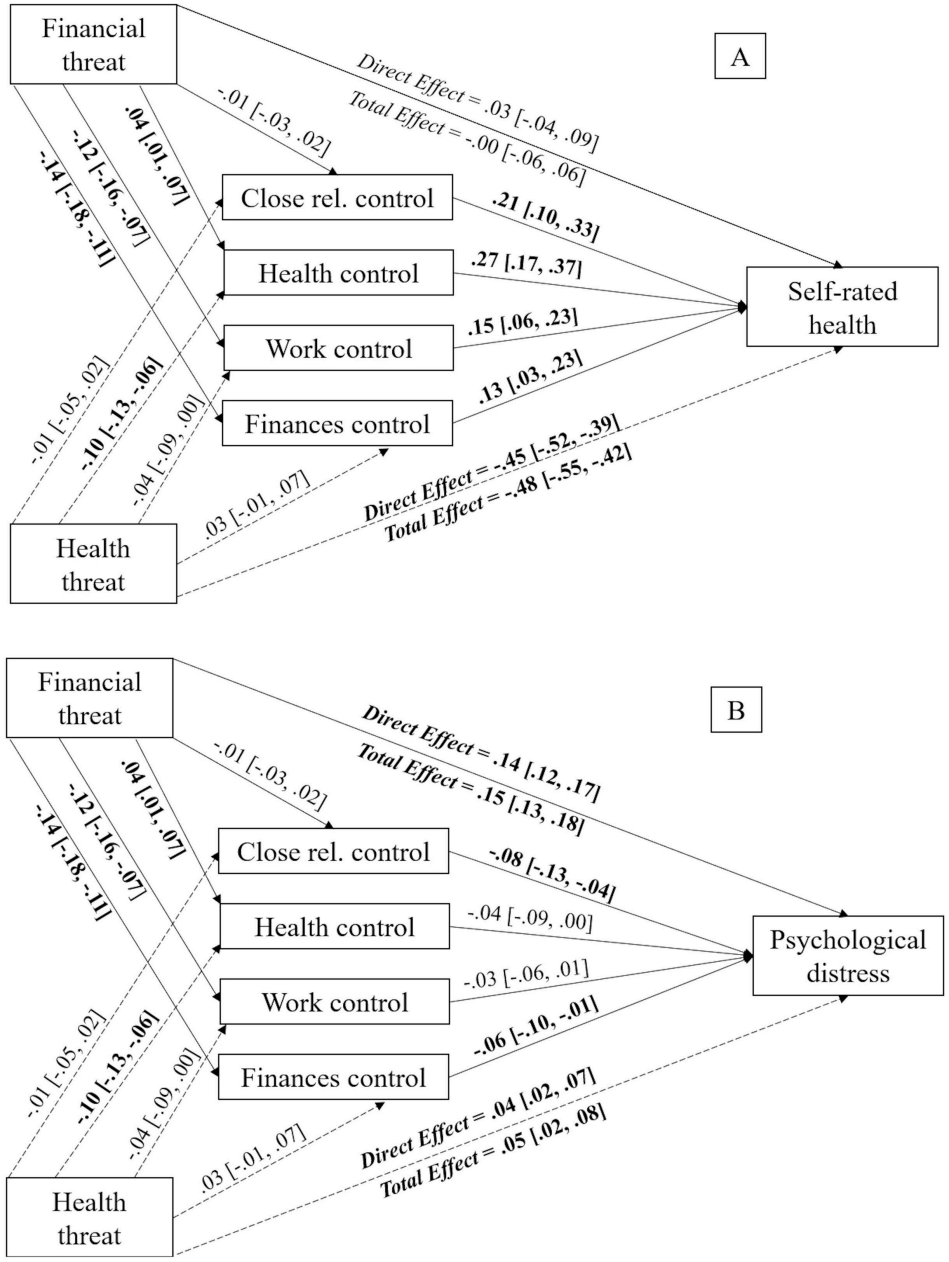
Mediation models displaying the indirect effects of perceived financial threat on self-rated health status **(A)** and psychological distress **(B)** via perceived personal control in different life domains with perceived health threat as covariate. Unstandardized estimates, with 95% CI reported between brackets. Significant effects appear in bold text. The bootstrap sample size was 10,000. Close rel., close relationships.

Continuing with self-rated health status, previous results were replicated again. Personal perceived personal control over health [*b* = 0.01, *SE* = 0.01, 95% CI (0.00, 0.02)], work [*b* = −0.02, *SE* = 0.01, 95% CI (−0.03, −0.01)] and finances [*b* = −0.02, *SE* = 0.01, 95% CI (−0.04, −0.00)] had a significant indirect effect. Nevertheless, the effect of perceived personal control over close relationships was not significant [*b* = −0.00, *SE* = 0.00, 95% CI (−0.01, 0.01)]. Moreover, the direct effect of perceived financial threat on self-rated health status stopped being significant [*b* = 0.03, *SE* = 0.03, 95% CI (−0.04, 0.08)] after the inclusion of the mediating variables (i.e., perceived personal control over finances), which leads us to affirm that this was a complete mediation. The remaining paths appear in Figure 4A.

Lastly, in regards to psychological distress, it was observed that only perceived personal control over finances [*b* = 0.01, *SE* = 0.00, 95% CI (0.00, 0.02)] had a mediating effect between perceived financial threat and psychological distress. On the other hand, perceived personal control over close relationships [*b* = 0.00, *SE* = 0.00, 95% CI (−0.00, 0.00)], over health [*b* = −0.00, *SE* = 0.00, 95% CI (−0.00, 0.00)] and over work [*b* = 0.00, *SE* = 0.00, 95% CI (−0.00, 0.01)] did not have any mediating effect. This was a partial mediation due to the direct effect of perceived financial threat on self-rated health status remaining significant after the inclusion of the mediating variables [*b* = 0.14, *SE* = 0.01, 95% CI (0.12, 0.17)]. The remaining paths can be found on Figure 4B.

In summary, our results showed that greater perceived financial threat predicted higher levels of perceived personal control over health and lower levels of perceived personal control over work and finances. In turn, these types of control predicted higher levels of satisfaction with life, happiness and self-rated health status. Moreover, for the case of perceived personal control over finances, it also predicted lower levels of psychological distress.

#### Perceived health threat, perceived personal control in different life domains, and wellbeing/psychological distress: testing the mediating role of perceived personal control in different life domains

To put Hypothesis 2d to the test we ran one parallel mediation analysis per indicator of subjective wellbeing, health, and psychological distress, considering perceived financial threat as a covariate in order to control for its effects, performing a total of 4 mediation analyses.

**Figure 5:**
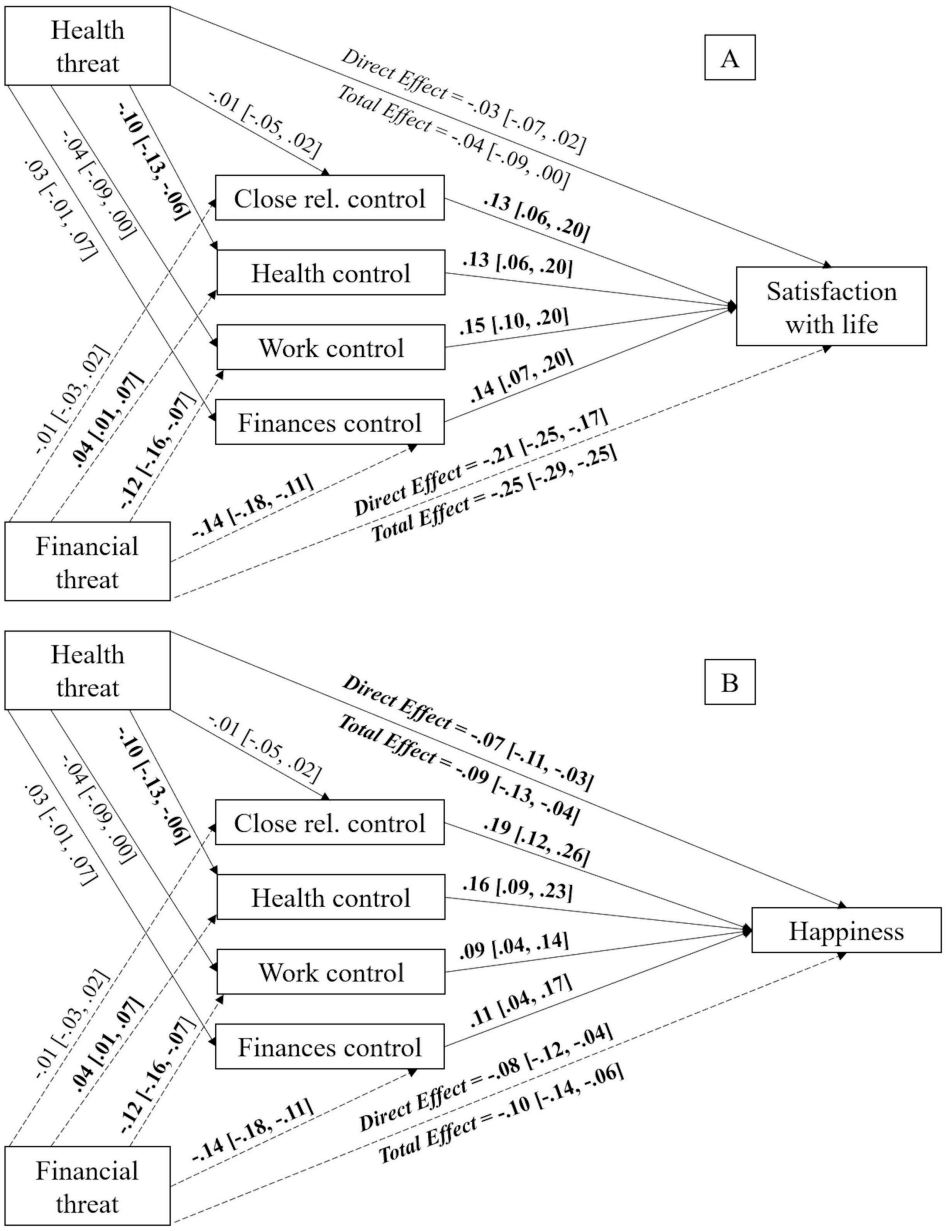
Mediation models displaying the indirect effects of perceived health threat on satisfaction with life **(A)** and happiness **(B)** via perceived personal control in different life domains with perceived financial threat as covariate. Unstandardized estimates, with 95% CI reported between brackets. Significant effects appear in bold text. The bootstrap sample size was 10,000. Close rel., close relationships.

For the case of satisfaction with life, perceived personal control over health [*b* = −0.01, *SE* = 0.00, 95% CI (−0.02, −0.00)] had a mediating effect on the relationship between perceived health threat and life satisfaction. However, perceived personal control over close relationships [*b* = −0.00, *SE* = 0.00, 95% CI (−0.01, 0.00)], work [*b* = −0.01, *SE* = 0.00, 95% CI (−0.01, 0.00)] and finances [*b* = 0.00, *SE* = 0.00, 95% CI (−0.00, 0.01)] did not. Since the direct effect of perceived health threat over satisfaction with life [*b* = −0.03, *SE* = 0.02, 95% CI (−0.07, −0.01)] was not statistically significant after the inclusion of the mediating variables, this mediation is considered a complete mediation. Figure 5A shows all remaining paths.

In regard to happiness, results showed that perceived personal control over health [*b* = −0.02, *SE* = 0.00, 95% CI (−0.03, −0.01)] had a mediating effect on the relationship between perceived health threat and happiness. However, perceived personal control over close relationships [*b* = −0.00, *SE* = 0.00, 95% CI (−0.01, 0.00)], work [*b* = −0.00, *SE* = 0.00, 95% CI (−0.01, 0.00)] and finances [*b* = 0.00, *SE* = 0.00, 95% CI (−0.00, 0.01)] did not have any mediating effect. The direct effect of perceived health threat on happiness [*b* = −0.07, *SE* = 0.02, 95% CI (−0.11, −0.03)] remained significant after the inclusion of the mediating variables, meaning this was a partial mediation, as shown in Figure 5B.

**Figure 6:**
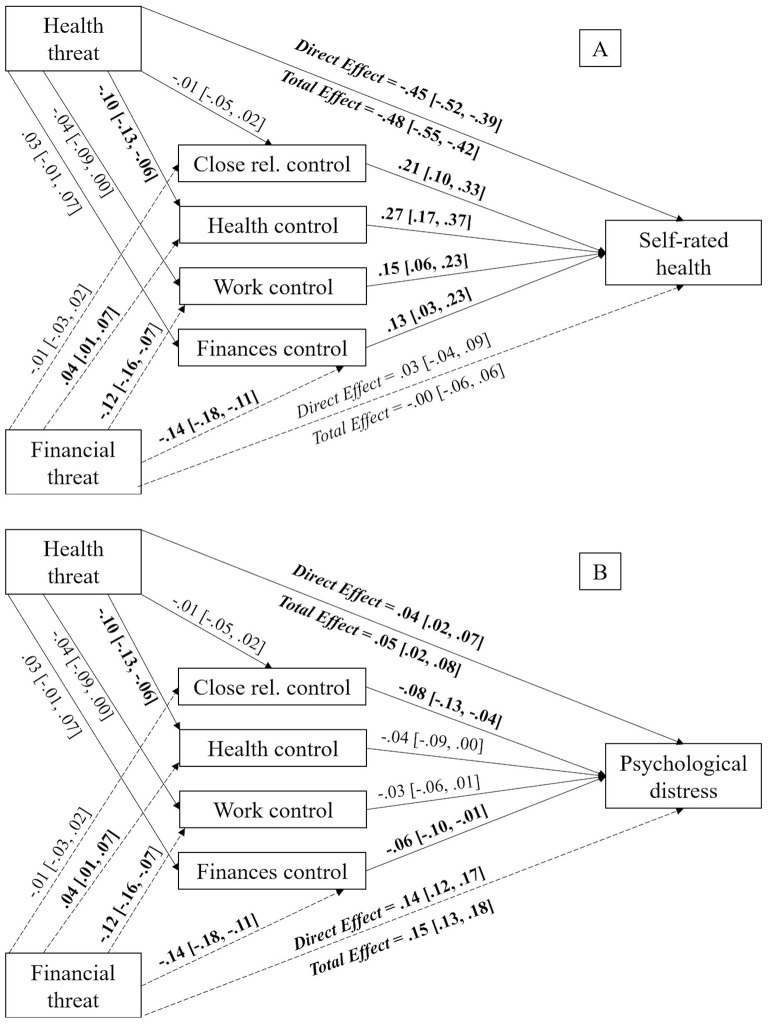
Mediation models displaying the indirect effects of perceived health threat on self-rated health status **(A)** and psychological distress **(B)** via perceived personal control in different life domains with perceived financial threat as covariate. Unstandardized estimates, with 95% CI reported between brackets. Significant effects appear in bold text. The bootstrap sample size was 10,000. Close rel., close relationships.

Regarding self-rated health status, perceived personal control over health [*b* = −0.03, *SE* = 0.01, 95% CI (−0.04, −0.01)] mediated the relationship between perceived health threat and self-rated health status. Nonetheless, perceived personal control over close relationships [*b* = −0.00, *SE* = 0.00, 95% CI (−0.01, 0.00)], work [*b* = −0.01, *SE* = 0.00, 95% CI (−0.02, 0.00)] and finances [*b* = −0.00, *SE* = 0.00, 95% CI (−0.00, 0.01)] did not mediate this relationship. This mediation was partial because the direct effect of perceived health threat on self-rated health status [*b* = −0.45, *SE* = 0.03, 95% CI (−0.52, −0.37)] remained significant after the inclusion of the mediating variables, as can be seen in Figure 6A.

Finally, when considering psychological distress, results indicated that perceived personal control over close relationships [*b* = 0.00, *SE* = 0.00, 95% CI (−0.00, 0.00)], health [*b* = 0.00, *SE* = 0.00, 95% CI (−0.00, 0.01)], work [*b* = −0.00, *SE* = 0.00, 95% CI (−0.00, 0.00)] and finances [*b* = −0.00, *SE* = 0.00, 95% CI (−0.01, 0.00)] did not have any mediating effect. Thus, perceived personal control in different life domains did not mediate the connection between perceived health threat and psychological distress, which can be observed in Figure 6B.

To sum up, our results showed that greater perceived health threat was indirectly related to reduced satisfaction with life, happiness, and self-rated health status via perceived personal control over health. More specifically, higher perceived health threat predicted lower levels of perceived personal control over health. In turn, these types of control predicted higher subjective wellbeing and health.

## Discussion

This research focused on examining the relationships between perceived financial and health threats and subjective wellbeing and health and how perceived personal control over different life domains influenced such relationships. Extant research has established that there is a relationship between perceived financial and health threats and subjective wellbeing and health, showing that during economic crises physical and mental health deteriorates (Navarro-Carrillo et al., 2020; Wang et al., 2020). Thus, during the COVID-19 pandemic a new context of instability arose, giving way to the experimentation of all of these negative consequences with a new factor added: the psychological experience of threat over health status (Moya and Willis, 2020).

Taking all of this into consideration, we carried out three independent studies with the intention to better comprehend the underlying mechanisms of the associations between the psychological experiences of financial and health threats and subjective wellbeing, self-rated health status, and psychological distress. Our proposed underlying mechanism was the mediating effect of perceived personal control in different life domains, which stems from the understanding that perceived personal control varies during life and also throughout different life domains (Lachman and Weaver, 1998a).

Study 1 confirmed our Hypothesis 1a: heightened worry over one's own financial situation was predictive of lower levels of satisfaction with life, happiness, and self-rated health status. To further apply this notion to the context of the COVID-19 pandemic and the relevance that worry over health had earned over that period, Study 2 replicated the same methods but, in this case, it evaluated the subjective perception of health threat. Once again, our hypothesis 1b was confirmed, with those participants who were more worried about their health status also exhibiting lower levels of subjective wellbeing and and perceiving that they had a worse health status. These effects arose even when controlling for the effects of the other source of perceived threat (i.e., perceived financial threat). Finally, pre-registered Study 3 took to replicate these findings and, in general, found the same effects except for perceived financial threat not predicting happiness, partially confirming Hypothesis 1c. Furthermore, all of these effects arose independently from sociodemographic characteristics and also independently from indicators of objective and subjective socioeconomic status. These findings are in line with recent literature, which highlights the strong negative connection between both perceived financial (Jha and Jha, 2022) and health threats (Wu et al., 2024) to subjective wellbeing.

Regarding perceived personal control, available evidence suggests that this variable is at risk during economic crises due to context instability (Fritsche et al., 2017), being able to mediate the relationship between fear of worsening of personal finances and psychosocial variables. Study 1 supported this notion, showing that perceived personal control in different life domains has the ability to mediate the effects between perceived financial threat and wellbeing and self-rated health status. More specifically, we found that higher levels of perceived financial threat predicted lower levels of perceived personal control over health and work. Simultaneously, these types of control predicted higher levels of satisfaction with life, happiness and self-rated health status, confirming, Hypothesis 2a. This may be due to the fact that the salience of health-related situations during the period on which the research was carried out influenced the results, putting the focus of worry and recovery of sense of agency on health aspects (Wu et al., 2024), while also considering the employment issues that were taking place in Spain. Furthermore, evidence suggests that perceived physical and mental health have a reciprocal relation to job insecurity, considered a form of financial threat. The experience of worse health heightens worries over job stability and vice-versa (Rudolph et al., 2025), so it would be understandable that the perception of heightened control over health and work would prevent the negative effects of perceived financial threat over subjective wellbeing.

Study 2 delved deeper into the relevance of this health-related stress and was centered around the psychological experience of heightened threat over the individual's own health status. This study revealed that higher levels of perceived health threat predicted lower levels of perceived personal control over close relationships and and health. At the same time, these types of control predicted higher levels of wellbeing and health, confirming Hypotheses 1b and 2b. This is supported by Li et al.'s (2020) research, which found that participants were quite affected by the disconnection they suffered from their loved ones during the pandemic periods of lockdown and also the asseverations made by Moya and Willis (2020), indicating that individuals were going to be severely affected by their forced separation from the people to whom they were closest. In addition to the justifications presented in Study 1 in regard to perceived personal control over health, studies carried out during the pandemic show that higher levels of sense of control were connected to higher levels of positive mental health (Capaldi et al., 2025). Furthermore, Wang et al. (2020) found that 75% of their sample were worried about their family members contracting COVID-19, which aligns with both life domains. Even though it cannot be confirmed here, it is likely that some suffering derived from the consequences of the high levels of alarm posed by possible infections. Not only that, but participants could have also suffered from COVID-19 or have lost a loved one during that time.

Pre-registered Study 3 not only sought out to replicate the findings of Studies 1 and 2 but also included a measure of psychological distress. Additionally, this study employed an improved sampling method which led to obtaining a more representative sample of the Spanish population. In general, the findings of this study were in line with previous hypotheses and findings. Mediation analysis showed that higher levels of perceived financial threat were connected to lower levels of subjective wellbeing and self-rated health through perceived personal control over health, work and finances. This confirms our initial hypothesis (2c) that perceived personal control over work and finances are connected to fears of financial instability. Also, the study highlights that perceived personal control over health continues to be a relevant mediator between psychological threat and wellbeing and health. These results are also in line with recent research that indicates that sense of control buffers the negative effect of perceived financial threat on employee wellbeing (Jha and Jha, 2022). Nevertheless, the link of perceived financial threat with psychological distress was only mediated by perceived personal control over finances, contradicting Hypothesis 2d. Regarding perceived health threat, and unlike in Study 2, only perceived personal control over health mediated the connection of this elevated source of threat to lower levels of subjective wellbeing and self-rated health, only partially confirming Hypothesis 2d. This could be caused by the temporalization of this study, with COVID-19 preventative measures not having been present for a year, meaning that participant had recovered close contact with their loved ones and workplaces had returned to a pre-pandemic situation, with no masks, no social distancing and a return to in-person work which rose social contact.

Importantly, this research is not without its limitations. It is important to highlight the fact that this is a correlational research and, because of that, no causal conclusions can be derived from it. Indeed, future research could focus on designing experimental studies in order to dive into the causality and inner mechanisms of the relationship between psychological experiences of threat and different wellbeing and health measures. These experiments could also serve to assess the directionality of the linkage between perceived threat and perceived personal control, in order to assure which phenomenon precedes the other. Moreover, each of the three studies presented the self-reported measures on a single survey, which could make the participants aware of the themes of the survey. Future research could avoid this by randomizing the presentation of the measures and including distracting tasks. Continuing with the content of the surveys, it is necessary to acknowledge the use of single-item measures for evaluating perceived personal control in different life domains as a limitation. This approach allowed the research to be carried out in a timely manner during the COVID-19 pandemic and to include the items in the survey for Study 3, which presented length limitations. However, the stated practicality, alongside prior research employing said items (Bishop, 2005), does not account for the potential decrease in reliability.

Further research could also add a multicultural aspect to these studies, testing whether other countries affected by financial or health crises show the same psychological patterns and taking into account other culture's conceptualization of perceived personal control. Buyukkecec (2021) shows the existence of different responses to the COVID-19 pandemic and Lachman et al. (2015) mention how sense of control in Americans is more tightly connected to subjective wellbeing than to other nationalities such as the Japanese.

Moreover, other types of psychological threat should be assessed to determine whether the mediating abilities of perceived personal control in different life domains are generalized to different types of perceived threat or if it is necessary to consider different domains regarding different types of perceived threat, such as perceived climate threat. To deeply understand the mechanisms underlying the linkage of psychological threat, perceived personal control and subjective wellbeing some qualitative data should be collected, in order for participants to be able to explain how they understand these connections. Another important addition would be a longitudinal study that could allow the assessment of the differences in perceived threat, perceived personal control and subjective wellbeing, health and psychological distress pre and post-pandemic.

Considering the findings of this research, some practical implications could be derived from it. With more evidence of the connection of psychological threat, control and wellbeing, organizations such as governments and companies could develop interventions or programs that lower levels of perceived threat and strengthen perceived personal control in different areas of life. For example, evidence implies that safety perceptions in the workplace are capable of enhancing employees wellbeing (Lee and Rhee, 2022), which could be extended to the implementation of measures to prevent financial and health difficulties. However, it is important to consider the complex nature of personal control: even though it is connected to higher levels of wellbeing, higher levels of personal control are also connected to greater perceptions of inefficacy (Zhen et al., 2023) and could lead to feelings of guilt (Lachman and Weaver, 1998b).

It is worth noting that the three studies presented here highlight the relevance that health, specifically mental health, gained during the COVID-19 pandemic. Evidence of the effects of perceived threat on subjective wellbeing has been stablished throughout the literature, but this pandemic brought forth numerous negative health outcomes (Lindberg et al., 2025; Wang et al., 2020). The surveys for Studies 1 and 2 did not mention the pandemic. Nevertheless, both researches were carried out during the presence of COVID-19 preventative measures and right after these were lifted. Study 3 was carried out in an almost post-pandemic period. As such, participants were either experiencing first-hand the effects of the pandemic or were reminded of it in order to activate perceptions of threat. However, it could be discussed that the results of these investigations are not constrained to the COVID-19 pandemic since they are aligned with findings from previous unstable periods (Navarro-Carrillo et al., 2021) and other types of psychological threat (Soutar and Wand, 2022).

In conclusion, the detrimental effects of perceived financial threat on subjective wellbeing, self-rated health and psychological distress are mediated by perceived personal control over health, work, and finances, highlighting the numerous life domains that are affected by this type of psychological threat. In the case of perceived health threat, close relationships and health are the domains of perceived personal control that mediate its connection to subjective wellbeing and self-rated health, emphasizing the relevance of social connections and the risk at which health was put during the pandemic crisis. Overall, these findings are in keeping with our proposal that perceived personal control is not actually a unitary variable but that it is, indeed, a more complex construct that can change depending on the area of life that is highlighted in different situations. Not only that, but it is a relevant mechanism to further understand the ways in which psychological threat and wellbeing and health are connected.

## Data Availability

The datasets analyzed in this study can be found in OSF, https://osf.io/jex6t.
