# Supplementary material for: Perceived financial and health threats and wellbeing: the role of personal control in different life domains
Source: Front Psychol. 2025 Oct 31;16:1539794. doi: 10.3389/fpsyg.2025.1539794 (PMC12615432; doi:10.3389/fpsyg.2025.1539794)
Supplement: Supplementary file 1 [file Table_1.docx]

Supplementary Material

**Title: Perceived financial and health threats and wellbeing: the role of personal control in different life domains**

**Authors: Canal-Serantes, B., Navarro-Carrillo, G. & Valor-Segura, I.**

**Table S1**. Sociodemographic data frequencies from Studies 1 and 2.

| Variable | Study 1 (*N* = 382) | | Study 2 (*N* = 723) | |
| --- | --- | --- | --- | --- |
|  | n | % | n | % |
| **Civil status** |  |  |  |  |
| Single | 72 | 18.8 | 389 | 53.8 |
| In a relationship | 59 | 15.4 | 258 | 35.7 |
| Non-married couples (or cohabiting) | 45 | 44.8 | 52 | 7.2 |
| Married | 172 | 45 | 21 | 2.9 |
| Divorced | 28 | 7.3 | 3 | .4 |
| Widowed | 6 | 1.6 | - | - |
| **Current employment situation** |  |  |  |  |
| Student | 41 | 10.7 | 561 | 77.6 |
| Full-time job | 231 | 60.5 | 57 | 7.9 |
| Part-time job | 53 | 13.9 | 58 | 8 |
| Retired | 19 | 5 | 3 | .4 |
| Unemployed | 38 | 9.9 | 44 | 6.1 |
| **Family income level** |  |  |  |  |
| <650€ | 17 | 4.5 | 64 | 8.9 |
| 651-1,300€ | 72 | 18.8 | 175 | 24.2 |
| 1,301-1,950€ | 92 | 24.1 | 167 | 31.1 |
| 1,951-3,250€ | 99 | 25.9 | 191 | 26.4 |
| 3,251-4,550€ | 55 | 14.4 | 85 | 11.8 |
| 4,551-5,200€ | 23 | 6 | 22 | 3 |
| 5,201-5,800€ | 9 | 2.4 | 5 | .7 |
| >5,800€ | 15 | 3.9 | 14 | 1.9 |
| **Educational attainment** |  |  |  |  |
| Primary school | 24 | 6.3 | 2 | .3 |
| Secondary education | 28 | 7.3 | 6 | .8 |
| Vocational training | 92 | 24.1 | 21 | 2.9 |
| Baccalaureate | 38 | 9.9 | 2 | .3 |
| University not completed | 44 | 11.5 | 434 | 60 |
| University completed | 113 | 29.6 | 49 | 6.8 |
| Master’s | 33 | 8.6 | 163 | 22.5 |
| Doctorate | 10 | 2.6 | 46 | 6.4 |
| **Occupation** |  |  |  |  |
| Technical occupations | 107 | 28 | 210 | 29 |
| Senior management | 19 | 5 | 13 | 1.8 |
| Office works | 66 | 17.3 | 30 | 4.1 |
| Salesperson | 33 | 8.6 | 19 | 2.6 |
| Profesional on the service sector | 62 | 16.2 | 55 | 7.6 |
| Skilled worker | 33 | 8.6 | 9 | 1.2 |
| Agricultural worker | 13 | 3.4 | 3 | .4 |
| Unemployed | 49 | 12.8 | 384 | 53.1 |

**Table S2**. Sociodemographic data frequencies from Study 3.

| Variable | Study 3 (*N* = 1541) | |
| --- | --- | --- |
|  | n | % |
| **Civil status** |  |  |
| Single | 438 | 28.4 |
| In a relationship | 1103 | 71.6 |
| **Family income level** |  |  |
| <650€ | 172 | 11.2 |
| 651-1,300€ | 291 | 18.9 |
| 1,301-1,950€ | 270 | 17.5 |
| 1,951-3,250€ | 524 | 34.0 |
| 3,251-4,550€ | 179 | 11.6 |
| 4,551-5,200€ | 43 | 2.8 |
| 5,201-5,800€ | 3 | .2 |
| >5,800€ | 59 | 3.8 |
| **Educational attainment** |  |  |
| No studies | 16 | 1.0 |
| Primary school | 31 | 2.0 |
| Two first years of high school | 155 | 10.1 |
| High school, baccalaureate, vocational training | 609 | 39.5 |
| Undergraduate | 244 | 15.8 |
| Graduate school | 292 | 18.9 |
| Master’s | 165 | 10.7 |
| Doctorate | 29 | 1.9 |

**Table S3**. Bivariate correlations, means, standard deviations and reliability of scales of Study 1.

|  | 1 | 2 | 3 | 4 | 5 | 6 | 7 | 8 | 9 | 10 | 11 | 12 | α |
| --- | --- | --- | --- | --- | --- | --- | --- | --- | --- | --- | --- | --- | --- |
| 1. Financial threat | - |  |  |  |  |  |  |  |  |  |  |  | .89 |
| 2. Income | -.22** | - |  |  |  |  |  |  |  |  |  |  |  |
| 3. Educational attainment | -.08 | .26** | - |  |  |  |  |  |  |  |  |  |  |
| 4. Occupation | .18** | .33** | -.42** | - |  |  |  |  |  |  |  |  |  |
| 5. SES | -.38** | .35** | .32** | -.31** | - |  |  |  |  |  |  |  |  |
| 6. Satisfaction with life | -.49** | .27** | .23** | -.22** | .54** | - |  |  |  |  |  |  | .87 |
| 7. Subjective Happiness | -.48** | .08 | -.04 | -.67 | .34** | .60** | - |  |  |  |  |  | .80 |
| 8. Self-perceived health | -.19** | .05 | .07 | -.12* | .15** | .32** | .38** | - |  |  |  |  |  |
| 9. Control over close relationships | -.10 | -.05 | -.07 | .03 | .11* | .23** | .23** | .21** | - |  |  |  |  |
| 10. Control over health | -.20** | -.05 | .02 | .03 | .17** | .25** | .32** | .42** | .21** | - |  |  |  |
| 11. Control over work | -.24** | .11* | -.05 | -.12* | .27** | .44** | .30** | .20** | .27** | .16** | - |  |  |
| 12. Control over finances | -.25** | .16** | -.00 | -.18** | .27** | .35** | .24** | .09 | .23** | .21** | .48** | - |  |
| M | 2.45 | 3.74 | 4.49 | 3.84 | 6.09 | 3.44 | 4.95 | 3.82 | 3.54 | 3.52 | 3.55 | 3.64 |  |
| SD | 1.01 | 1.60 | 1.83 | 2.40 | 1.52 | .89 | 1.20 | .94 | .91 | .99 | 1.11 | 1.13 |  |

*Note.* * *p <* .05, ** *p <* .01. SSC = Subjective socioeconomic status

**Table S4**. Bivariate correlations, means, standard deviations and reliability of scales of Study 2

|  | 1 | 2 | 3 | 4 | 5 | 6 | 7 | 8 | 9 | 10 | 11 | 12 | 13 | α |
| --- | --- | --- | --- | --- | --- | --- | --- | --- | --- | --- | --- | --- | --- | --- |
| 1. Health threat | - |  |  |  |  |  |  |  |  |  |  |  |  | .88 |
| 2. Financial threat | .66** | - |  |  |  |  |  |  |  |  |  |  |  | .88 |
| 3. Income | -.17** | -.24** | - |  |  |  |  |  |  |  |  |  |  |  |
| 4. Educational attainment | -.07* | -.07* | -.02 | - |  |  |  |  |  |  |  |  |  |  |
| 5. Occupation | .08* | .11** | -.12** | -.24** | - |  |  |  |  |  |  |  |  |  |
| 6. SES | -.22** | -.39** | .38** | .16** | -.14** | - |  |  |  |  |  |  |  |  |
| 7. Satisfaction with life | -.36** | -.41** | .14** | .16** | -.14** | .44 | - |  |  |  |  |  |  | .87 |
| 8. Subjective Happiness | -.43** | -.44** | .11** | .16** | -.17** | .35** | .67** | - |  |  |  |  |  | .86 |
| 9. Self-perceived health | -.38** | -.25** | .18** | .12** | -.12** | .25** | .40** | .44** | - |  |  |  |  |  |
| 10. Control over close relationships | -.21** | -.20** | .05 | .09* | -.04 | .16** | .36** | .36** | .21** | - |  |  |  |  |
| 11. Control over health | -.28** | -.20** | .04 | .07 | -.06 | .19** | .28** | .32** | .44** | .16** | - |  |  |  |
| 12. Control over work | -.16** | -.16** | .02 | .06 | -.12** | .19** | .26** | .27** | .23** | .27** | .30** | - |  |  |
| 13. Control over finances | -19** | -.24** | .07 | .09* | -.15** | .24** | .30** | .26** | .21** | .16** | .30** | .34** | - |  |
| M | 2.57 | 2.74 | 3.3 | 5.61 | 5.29 | 5.61 | 3.15 | 4.38 | 3.78 | 3.35 | 3.41 | 3.16 | 2.76 |  |
| SD | 1.01 | 1.03 | 1.45 | 1.19 | 3.12 | 1.45 | .92 | 1.35 | .83 | .82 | .89 | 1.06 | 1.13 |  |

*Note.* * *p <* .05, ** *p <* .01. SCC = Subjective socioeconomic status

**Table S5**. Bivariate correlations, means, standard deviations and reliability of scales for Study 3.

|  | 1 | 2 | 3 | 4 | 5 | 6 | 7 | 8 | 9 | 10 | 11 | 12 | 13 | 14 | 15 | α |
| --- | --- | --- | --- | --- | --- | --- | --- | --- | --- | --- | --- | --- | --- | --- | --- | --- |
| 1. Health threat | - |  |  |  |  |  |  |  |  |  |  |  |  |  |  | .83 |
| 2. Financial threat | .44^**^ | - |  |  |  |  |  |  |  |  |  |  |  |  |  | .75 |
| 3. Family income | -.07** | -.25** | - |  |  |  |  |  |  |  |  |  |  |  |  |  |
| 4. Educational attainment | -.11** | -.16** | -.26** | - |  |  |  |  |  |  |  |  |  |  |  |  |
| 5. Income SES | -.09^**^ | -.37^**^ | .31** | .26** | - |  |  |  |  |  |  |  |  |  |  |  |
| 6. Educational attainment SES | -.11^**^ | -.16^**^ | .19** | .55** | .41^**^ | - |  |  |  |  |  |  |  |  |  |  |
| 7. Occupational status SES | -.12^**^ | -.33^**^ | .27** | .25** | .61^**^ | .48^**^ | - |  |  |  |  |  |  |  |  |  |
| 8. Satisfaction with life | -.19^**^ | -.34^**^ | .17** | .08** | .37^**^ | .16^**^ | .36^**^ | - |  |  |  |  |  |  |  | .88 |
| 9. Subjective Happiness | -.17^**^ | -.19^**^ | .06* | -.02 | .22^**^ | .10^**^ | .24^**^ | .61^**^ | - |  |  |  |  |  |  | .83 |
| 10. Self-rated health | -.37^**^ | -.16^**^ | .04 | .02 | .22^**^ | .11^**^ | .21^**^ | .43^**^ | .43^**^ | - |  |  |  |  |  |  |
| 11. Pysch. distress | .22^**^ | .34^**^ | -.12** | .01 | -.21^**^ | -.05^*^ | -.24^**^ | -.44^**^ | -.56^**^ | -.42^**^ | - |  |  |  |  |  |
| 12. Control over close relationships | -.03 | -.02 | -.03 | -.02 | .06^*^ | .08^**^ | .10^**^ | .21^**^ | .25^**^ | .20^**^ | -.15^**^ | - |  |  |  |  |
| 13. Control over health | -.11^**^ | .01 | -.05 | -.07* | .09^**^ | -.01 | .09^**^ | .22^**^ | .25^**^ | .26^**^ | -.13^**^ | .35^**^ | - |  |  |  |
| 14. Control over work | -.12^**^ | -.18^**^ | .08** | .05* | .25^**^ | .12^**^ | .31^**^ | .30^**^ | .24^**^ | .23^**^ | -.18^**^ | .32^**^ | .28^**^ | - |  |  |
| 15. Control over finances | -.06^*^ | -.21^**^ | .04 | -.04 | .25^**^ | .05 | .22^**^ | .30^**^ | .26^**^ | .21^**^ | -.20^**^ | .33^**^ | .40^**^ | .45^**^ | - |  |
| M | 4.48 | 4.73 | 3.44 | 4.76 | 4.91 | 6.37 | 5.47 | 4.18 | 4.81 | 6.36 | 1.9 | 3.66 | 3.49 | 3.13 | 3.46 |  |
| SD | 1.51 | 1.68 | 1.58 | 1.37 | 1.51 | 1.86 | 1.96 | 1.31 | 1.23 | 2.00 | .87 | .89 | .99 | 1.25 | 1.07 |  |

*Note.* * *p <* .05, ** *p <* .01. SES = Subjective socioeconomic status. Psych = Psychological
